# Pathogen infection induces specific transgenerational modifications to gene expression and fitness in *Caenorhabditis elegans*

**DOI:** 10.3389/fphys.2023.1225858

**Published:** 2023-09-22

**Authors:** Phillip Wibisono, Jingru Sun

**Affiliations:** Department of Translational Medicine and Physiology, Elson S. Floyd College of Medicine, Washington State University, Spokane, WA, United States

**Keywords:** epigenetics, transgenerational modification, immunity, *C. elegans*, pathogen infection, stress response

## Abstract

How pathogen infection in a parental generation affects response in future generations to the same pathogen via epigenetic modifications has been the topic of recent studies. These studies focused on changes attributed to transgenerational epigenetic inheritance and how these changes cause an observable difference in behavior or immune response in a population. However, we questioned if pathogen infection causes hidden epigenetic changes to fitness that are not observable at the population level. Using the nematode *Caenorhabditis elegans* as a model organism, we examined the generation-to-generation differences in survival of both an unexposed and primed lineage of animals against a human opportunistic pathogen *Salmonella enterica*. We discovered that training a lineage of *C. elegans* against a specific pathogen does not cause a significant change to overall survival, but rather narrows survival variability between generations. Quantification of gene expression revealed reduced variation of a specific member of the TFEB lipophagic pathway. We also provided the first report of a repeating pattern of survival times over the course of 12 generations in the control lineage of *C. elegans*. This repeating pattern indicates that the variability in survival between generations of the control lineage is not random but may be regulated by unknown mechanisms. Overall, our study indicates that pathogen infection can cause specific phenotypic changes due to epigenetic modifications, and a possible system of epigenetic regulation between generations.

## 1 Introduction

Responding and adapting to environmental pressures is key to the survival of a species and a core concept of natural selection (Gregory, 2009). Natural selection is the slow process of increasing the proportion of beneficial and heritable characteristics from one generation to the next. This enrichment of beneficial traits in reproductive members of a population requires constant selective pressure from the environment over hundreds if not thousands of generations (Blount et al., 2008; Orr, 2009; Knöppel et al., 2018). While changing beneficial allele frequency in a population is necessary for the continued evolution of a species, it cannot respond to a rapid transient selection pressure, or counterintuitively, be slowed by the addition of a strong selection pressure (Ueda et al., 2017). These issues pose a problem for most organisms as most inhabit a dynamic environment that experience temporally isolated selection events such as the spread of a recurring communicable disease. To compensate for these issues, modifications to the gene expression regulatory system and the mechanisms that guide this regulation, known as epigenetics, provide organisms an inheritable, rapid, and reversible means of responding to rapid environmental pressures (Lacal and Ventura, 2018). Epigenetic changes can be transferred over multiple successive generations in a phenomenon known as transgenerational epigenetic inheritance (TEI) (Moore et al., 2019). TEI has been implicated in pathogen avoidance responses, transposon silencing, embryo development, and physiological responses to cellular stressors such as starvation, heat, or osmotic pressure in model organisms (Ghildiyal and Zamore, 2009; Rodgers et al., 2013; Rechavi et al., 2014; Siklenka et al., 2015; Burton et al., 2017; Klosin et al., 2017; Moore et al., 2019; Lismer et al., 2020). This epigenetic inheritance has also been linked to metabolic and adult-onset disease in humans due to endocrine disruptors, diet, and stress (Anway et al., 2006; Pembrey et al., 2014; Sales et al., 2017). These studies point to TEI as a key player in determining the fitness and success of a species.

TEI has also been associated with another phenomenon found in insects known as transgenerational immune priming (TGIP), where exposure to pathogens on either the maternal or paternal side can increase survival of offspring to the same pathogen (Gegner et al., 2019). TGIP involves either the transmission of bacteria, RNAs, or antimicrobial peptides to developing embryos, or histone modification of immune-related genes (Vilcinskas, 2021). However, these studies often only examine intergenerational transmission after pathogen exposure or specifically try to account for epigenetic changes in their data (Ferro et al., 2019; Tetreau et al., 2020). The longer-term effects of pathogen exposure on an organism multiple generations removed from the initial infection is still not well understood. This is in part possibly due to experiment focusing on longer term outcomes would be cumbersome and exhausting. For example, the common insect model organisms *Drosophila melanogaster* has a generation time of 9–10 days and a median lifespan of 70 days, an experiment measure 4 generations would take a minimum of 40 days and up to 110 days to complete (Fernández-Moreno et al., 2007; Piper and Partridge, 2018). Fortunately, TGIP is not exclusive to insects and has been observed in another common model organism, the nematode *C. elegans. C. elegans* has already been established as a model for studying TEI and is an optimal model organism for studying long term TGIP, due to its shorter generation time of 3 days, large brood size, conserved epigenetic mechanisms, and hermaphroditic reproduction system (Corsi et al., 2015; Weinhouse et al., 2018; Willis et al., 2021). *C. elegans* also does not require maternal care, and its embryos can be harvested from gravid adults aseptically, which removes the confounding variable of conditioning the animals against infection during their development (Stiernagle, 2006; Anyanful et al., 2009).

As mentioned previously, *C. elegans* has been used to study the effects of environmental insults on epigenetic inheritance and TGIP to specific pathogens (Jobson et al., 2015; Moore et al., 2019; Baugh and Hu, 2020). Examples of TGIP occurring in *C. elegans* are the inheritance of antiviral defense through RNA silencing, the production of PIWI-interacting RNAs, and the methylation of adenine N^6^ which promotes stress gene expression (Fire et al., 1998; Lu et al., 2005; Rechavi et al., 2011; Belicard et al., 2018; Ma et al., 2019; Gabaldón et al., 2020). These changes in gene expression of *C. elegans* have been shown to last up to 20 generations after the initial stimuli (Ashe et al., 2012). However, since *C. elegans* in the wild inhabits an environment with a diverse culture of pathogenic and non-pathogenic bacteria, it would be impractical and resource intensive to prime future offspring against every encountered pathogen (Schulenburg and Félix, 2017). Silencing gene expression in a diverse environment may also be detrimental to survival as knockout experiments in genes such as *npr-1* and *nmur-1* show contrasting survival phenotypes when the animals are challenged with different pathogens (Nakad et al., 2016; Wibi et al., 2022). In order to optimize its fitness in the wild, *C. elegans* would need to adjust its defense response in minute increments and allow for a greater degree of flexibility between generations.

It is clear that TGIP occurs in *C. elegans* and the nematode is able to adjust its defense response through a variety of means. Previous studies focusing on the mechanisms behind TEI and TGIP in *C. elegans* compared the unprimed and primed populations, and the differences between these groups over time. What these previous works may have overlooked by solely comparing the two populations are how *C. elegans* of a single lineage may naturally adjust its survival strategies over the course of multiple generations. In this study, we found that both the survival time of unprimed animals exposed to *Salmonella enterica* and the expression of antimicrobial genes fluctuate between generations, phenotypes not seen in other studies focusing on *S. enterica* survival (Aballay et al., 2000; Haskins et al., 2008; Styer et al., 2008; Sellegounder et al., 2019). However, when an offshoot of the same population of animals is primed against *S. enterica,* the oscillation in survival and gene expression are suppressed. These results suggest that TGIP not only narrows the variability in survival and gene expression against specific pathogens, but also that the survival and gene expression of unprimed populations can follow a recurring pattern. Overall, our study has uncovered a blind spot in using *C. elegans* to study TEI and TGIP and provided better insights into how organisms can adjust defense responses between generations.

## 2 Methods

### 2.1 Nematode strains

Wild-type *Bristol N2 C. elegans* were maintained as hermaphrodites at 20°C, grown on modified Nematode Growth Media (NGM) (0.35% instead of 0.25% peptone), and fed *Escherichia coli* OP50 (Brenner, 1974). A parent stock of wild-type *Bristol N2* animals was generated by growing a population on 40 10 cm NGM plates seeded with *E. coli* OP50 until starvation. The newly starved animals were collected using M9 buffer and transferred to a 50 mL conical tube. The animals were pelleted via centrifugation at 1,000 *×* *g* for 3 min and the supernatant removed. The pellet was resuspended in 40 mL of sterile S-buffer +15% (v/v) glycerol, the resuspended animals were separated into 1 mL aliquots and stored at −80°C.

### 2.2 Bacteria strains

The following bacteria strains were grown using standard conditions (Lagier et al., 2015): *Escherichia coli* strain OP50 and *S. enterica* strain SL1344. *E. coli* OP50::GFP was also cultured using standard conditions with the inclusion of 100 µg/mL of ampicillin.

### 2.3 Pathogen exposure

A new frozen stock of wild-type *Bristol N2* was recovered from −80°C for each lineage and allowed to grow at 20°C on NGM seeded with *E. coli* OP50 for two generations. Well-fed adult wild-type animals were collected using M9 buffer and centrifuge at 1,000 *g* for 3 min to form a worm pellet. The supernatant was removed, and the animals were lysed using 500 µL a solution consisting of 5 mL of 1N sodium hydroxide and 2 mL of an 8.25% (w/v) sodium hypochlorite solution for a total volume of 7 mL (Final concentrations of the lysis solution are 0.71N sodium hydroxide and 2.36% (w/v) sodium hypochlorite). The animals were gently agitated at room temperature for 4 min or until complete breakage of the adult animals was observed under a dissecting microscope. The lysis solution was diluted using 10 mL of M9 buffer and a pellet was formed by centrifugation at 1,000 *×* *g* for 3 min. The supernatant was removed and an additional 10 mL of M9 buffer was added to wash the released eggs, a total of 3 M9 washes were performed. The eggs were synchronized for 22 h in sterile S-buffer + 5 µg/mL of cholesterol at room temperature. Synchronized L1 larval animals were transferred onto modified NGM plates seeded with *E. coli* OP50 and allowed to grow at 20°C for 48 h. *E. coli* and *S. enterica* lawns were prepared by culturing the bacteria for 15∼16 h in Luria Broth (LB) at 37°C in a shaking incubator set to 200RPM. *E. coli* OP50::GFP was also prepared by culturing the bacteria in LB plus 100 µg/mL of ampicillin for 15∼16 h at 37°C in a shaking incubator set to 200RPM. 500 µL of liquid bacteria cultures were seeded onto 10 cm NGM plates with or without ampicillin and spread aseptically to form a large lawn. Plates were allowed to grow at 37°C for 24 h to form a thick bacterial lawn. After incubation the plates were allowed to cool at room temperature for at least 30 min. L4 larval animals were collected using M9 buffer and split into two separate populations. The two populations of animals were placed onto room temperature plates seeded with either *E. coli* OP50 or *S. enterica* SL1344, the two populations were then placed into the same 20°C incubator for 8 h (initial P_o_ generation). After 8 h, the animals were washed three times in M9 buffer containing 100 µg/mL of kanamycin before being placed onto NGM plates containing 100 µg/mL of ampicillin, seeded with *E. coli* OP50::GFP to hamper any *S. enterica* growth. The animals were allowed to grow until they reached 72 h old, at which time the animals were lysed using the previously mentioned method to prepare next-generation. The F_1_ through F_12_ generations in both lineages were grown on NGM plates seeded with *E. coli* OP50, neither lineage was exposed to *S. enterica* SL1344 after the initial P_o_ exposure.

### 2.4 Survival assay

Animals of both lineages were synchronized using the previously mentioned sodium hydroxide and sodium hypochlorite method and allowed to hatch at room temperature for 22 h in S-buffer with 5 µg/mL of cholesterol. The synchronized L1 larval animals were allowed to grow at 20°C for 72 h on fresh NGM seeded with *E. coli* OP50. The synchronized 72-h old adult animals from both the naïve and trained lineages were placed onto 3.5 cm NGM plates seed with *S. enterica* SL1344. Three *S. enterica* SL1344 NGM plates were prepared for each lineage with a 30 µL drop of fresh *S. enterica* SL1344 liquid bacteria culture which was grown in LB for 15∼16 h at 37°C in a shaking incubator set to 200RPM. The plates were gently swirled to create an approximately 1.5 cm in diameter lawn in the center of the plate. The seeded NGM plates were grown in a 37°C incubator for 15∼16 h. After incubation, plates were allowed to cool to room temperature for at least 30 min before the 72-h old animals were placed on the plates. 20 animals were placed onto each *S. enterica* SL1344 NGM plates for a total of 60 animals per lineage per assay. The survival assays were incubated at 20°C in a dedicated incubator and live animals were transferred daily to fresh plates until egg laying ceased. Animals were scored once a day and were considered dead when they failed to respond to touch.

### 2.5 RNA interference

RNA interference was conducted by feeding *C. elegans* with *E. coli* strain HT115(DE3) expressing double-stranded RNA (dsRNA) that is homologous to the target gene of interest. Briefly, *E. coli* with the appropriate vectors was grown in LB broth containing ampicillin (100 μg/mL) at 37 °C overnight, and plated onto NGM plates containing 100 μg/mL ampicillin and 3 mM isopropyl β-D-thiogalactoside (IPTG). RNAi-expressing bacteria were allowed to grow overnight at 37 °C. L2 and L3 larvae animals were placed on the fresh RNAi-expressing bacteria lawns and allowed to develop into gravid adults over 2 days at 20°C. Gravid adults were then transferred to fresh RNAi-expressing bacterial lawns and allowed to lay eggs at 25°C to generate a synchronized RNAi population. After 1 h, the gravid adults were removed from the plate and the eggs were allowed to develop into young adults over 65 h at 20°C. The young adults were then transferred to survival assay plates. Clone identity was confirmed by sequencing at Eton Bioscience Inc. *unc-22* RNAi was included as a positive control in all experiments to account for RNAi efficiency.

### 2.6 RNA collection and isolation

P_o_ 56-h old animals were collected after an 8-h exposure to either *E. coli* OP50 or *S. enterica* SL1344 at 20°C. The animals were washed three times with 10 mL of M9 buffer and centrifuge at 1,000 *×* *g* for 3 min to remove any excess bacteria. The supernatant was removed after the final M9 buffer wash and 400 µL of QIAzol (Qiagen) was added to the worm pellet. The samples were submerged in a bath of ethanol and dry ice immediately after the addition of QIAzol. F_1_ through F_12_ generations were collection followed a similar method, synchronized L1 larval animals were allowed to grow at 20°C on NGM plates seeded with *E. coli* OP50 until 56 h old before washing and collection in QIAzol. All RNA samples were stored at −80°C. RNA was isolated using the RNeasy Universal Plus Mini kit (Qiagen) following protocol provided by the manufacturer.

### 2.7 Quantitative real-time PCR (qRT-PCR)

Total RNA was obtained as described above. 2 µg of RNA were used to generate cDNA per 100 µL reaction using the Applied Biosystems High-Capacity cDNA Reverse Transcription Kit. qRT-PCR was conducted by following the prescribed protocol for PowerUp SYBR Green (Applied Biosystems) on a Bio-Rad CFX384 Touch real-time PCR machine (Bio-Rad). 10 µL reactions were set up following the manufacturer’s recommendations, and 20 ng of cDNA was used per reaction. Relative fold-changes for transcripts were calculated using the comparative *C*_*T*_(2^−ΔΔCT^) method and were normalized to pan-actin (*act-1, -3, -4*). Amplification cycle thresholds were determined by the CFX manager software. All samples were run in triplicate. Primer sequences used for this research are the following:

Pan-actin (*act-1, -3, -4*):

Forward 5’—TCG​GTA​TGG​GAC​AGA​AGG​AC—3’

Reverse 5’—CAT​CCC​AGT​TGG​TGA​CGA​TA—3’

*lipl-1*:

Forward 5’—GTT​TGT​GAC​GAT​GTG​ATG​TTC​C—3’

Reverse 5’—AAG​TTC​CTG​CGG​GTG​TAT​G—3’

*lipl-3*:

Forward 5’—CTG​TAC​TGG​AGT​GAT​GCA​GAT​T—3’

Reverse 5’—GAA​GTA​GTT​GTT​CTG​CGC​AAT​TAT -3’

*gst-4*:

Forward 5’—GAT​ACT​TGG​CAA​GAA​AAT​TTG​GAC—3’

Reverse 5’—TTG​ATC​TAC​AAT​TGA​ATC​AGC​GTA​A—3’

### 2.8 Quantification and statistical analysis

Survival curves were plotted using GraphPad PRISM (version 10) computer software. Survival was considered significantly different from the appropriate control indicated in the main text when *p* < 0.05. PRISM uses the product limit or Kaplan-Meier method to calculate survival fractions and the log-rank test, which is equivalent to the Mantel-Haenszel test. The TD_50_ for each survival curve was calculated using the area under the curve function and setting the *y*-axis baseline to 50 or “50% survival”. The resulting calculated *x*-axis value or “survival time” was used to plot the survival trend between generations. Statistical details for each figure are listed in its corresponding figure legend.

## 3 Results

### 3.1 *S. enterica* exposure during development suppresses survival variability in subsequent generations of *C. elegans*

**FIGURE 1:**
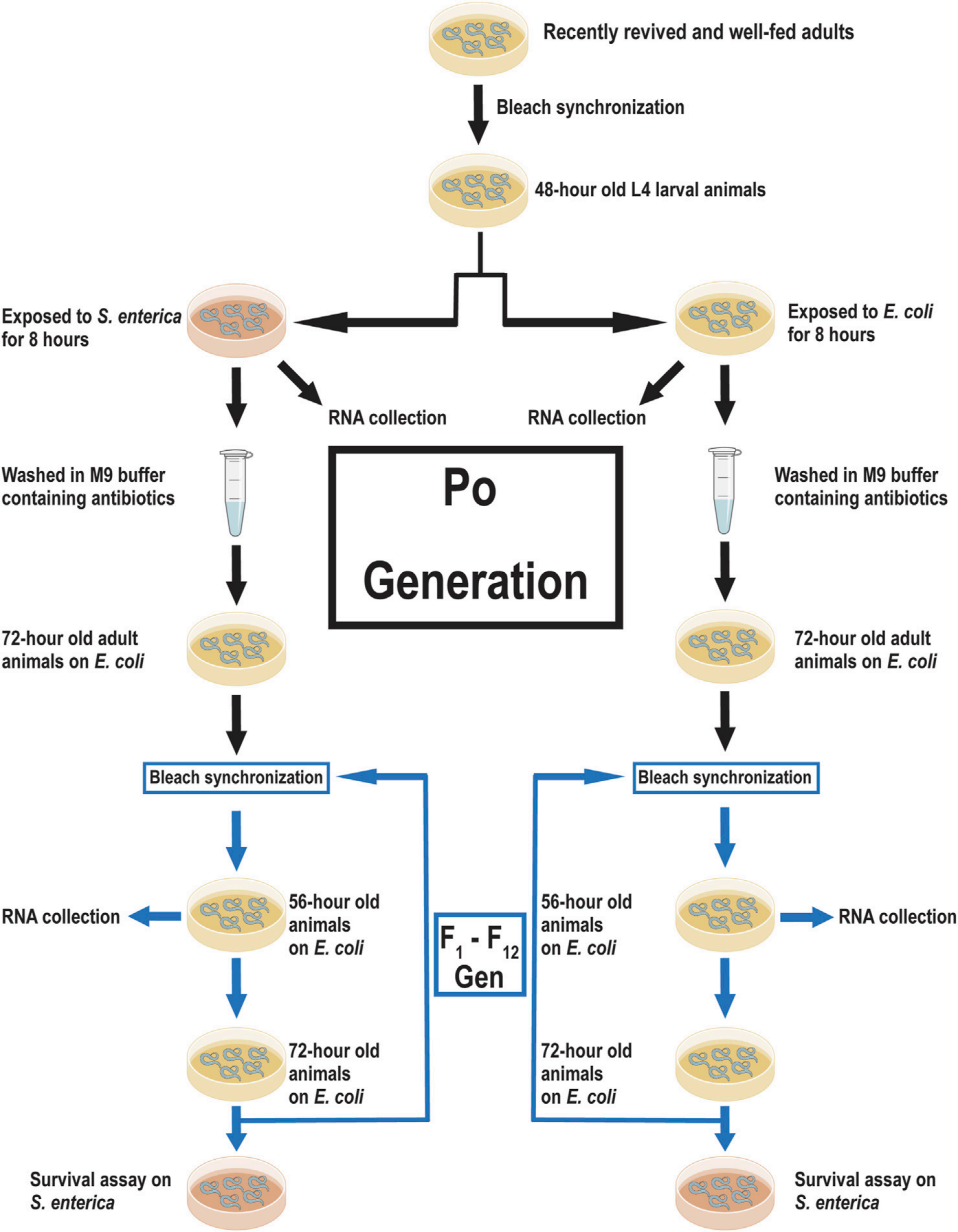
Experimental scheme for training *C. elegans* against *S. enterica* and data acquisition. A scheme of priming *C. elegans* against *S. enterica,* collection of RNA, and measuring survival time. Recently revived and well-fed adult wild-type animals were lysed to obtain a synchronized progenitor population. The synchronized progenitor population was allowed to grow to the L4 larval stage before being split into two lineages; naïve (*E. coli* OP50) and trained (*S. enterica* SL1344). RNA for the P_o_ generation was collected immediately after 8 h of incubation on either *E. coli* or *S. enterica*. The remaining members of these P_o_ populations were washed with antibiotics and allowed to develop into gravid adults. The two lineages were lysed and the next-generation was synchronized and allowed to grow on *E. coli* at 20°C. After 56 h, a sample of each lineage was collected for RNA isolation. When the animals reach 72-h old, individuals from each lineage were transferred to *S. enterica* seeded plates and survival time was measured. Animals were lysed at 72-h old and the subsequent generations were synchronized. 12 generations were collected for RNA isolation and measured for survival against *S. enterica*.

Pathogen exposure during L4 larval pre-adult development in *C. elegans* has been shown to cause transgenerational effects on subsequent generations (Rechavi et al., 2011; Sterken et al., 2014; Ma et al., 2019; Gabaldón et al., 2020). Offspring of animals exposed to members of the *Pseudomonas* family exhibited an enhanced avoidance behavior*,* increased dauer formation, or an increased expression of stress response genes (Palominos et al., 2017; Moore et al., 2019; Burton et al., 2020). However, these studies either did not measure survival over multiple generations or exposed multiple generations of offspring to the training pathogen before measuring survival. We sought to determine if a single prolonged exposure during the L4 larval stage would alter offspring survival against a slow killing pathogen over multiple generations. First, we exposed half a population of synchronized wild-type L4 larval animals to *Escherichia coli* OP50 and the other half to *S. enterica* SL1344 for 8 h to generate both naïve (*E. coli* OP50) and trained (*S. enterica* SL1344) lineages. *S. enterica* was selected as the training pathogen due to its low ability to invoke an avoidance response in *C. elegans* over 12 h, its lack of toxin production, and its predictability to slowly kill *C. elegans* via intestinal colonization over the course of 11 days as compared to 4 days using *Pseudomonas aeruginosa* (Sun et al., 2011; Sun et al., 2012; Sellegounder et al., 2018; Burton et al., 2020; Wibi et al., 2022). After exposure, the parental animals were washed to remove all bacteria and allowed to develop into gravid adults on *E. coli* OP50::GFP in the presence of 100 µg/mL of ampicillin to prevent *S. enterica* contamination. The animals were then lysed to obtain the F_1_ generation offspring. Subsequent generations (F_1_—F_12_) of both trained and naïve lineages were maintained on *E. coli* and were not exposed to *S. enterica* during development. F_1_ to F_12_ animals from both the trained and naïve lineages were transferred after 72 h to plates seeded with a partial lawn of *S. enterica* and the survival time was measured (Figure 1).

Seven different replicates were measured using this method, each replicate was established by thawing a new tube of frozen wild-type animals from the −80°C and allowing the animals to grow for two generations on *E. coli* OP50. All the replicates descend from a single large population of animals to account for any previously unknown genetic or epigenetic changes in the population. All replicates were maintained in the same dedicated incubator to account for fluctuations in temperature and humidity. Animals were synchronized by removing all bacteria and live animals using a combination of sodium hydroxide and sodium hypochlorite, and allowing the newly collected eggs to hatch in sterile S buffer with 5 µg/mL cholesterol. Larval L1 animals in the absence of food, halt development and allow for easier synchronization of the two lineages. All eggs which did not hatch in the allotted 22 h were removed to prevent poor synchronization. The naïve and trained lineages of a replicate were maintained on the same fresh batch NGM plates and seeded with *E. coli* OP50 from the same single colony. Both lineages of a replicate were washed with buffer from the same batch and all antibiotics were premeasured and aliquoted to account for any differences in drug exposure. All survival assays between the naïve and trained lineages shared the same *S. enterica* broth grown from a single colony. Both lineages were removed from the incubator during survival assay measurement to account for difference in temperature at the bench *versus* the incubator. The initial replicate was performed alone, while the other six replicates were performed in pairs. It is possible that differences between the naïve and trained lineages may have been caused by an unknown variable in the environment, the methods used attempted to minimize these outside influences.

**FIGURE 2:**
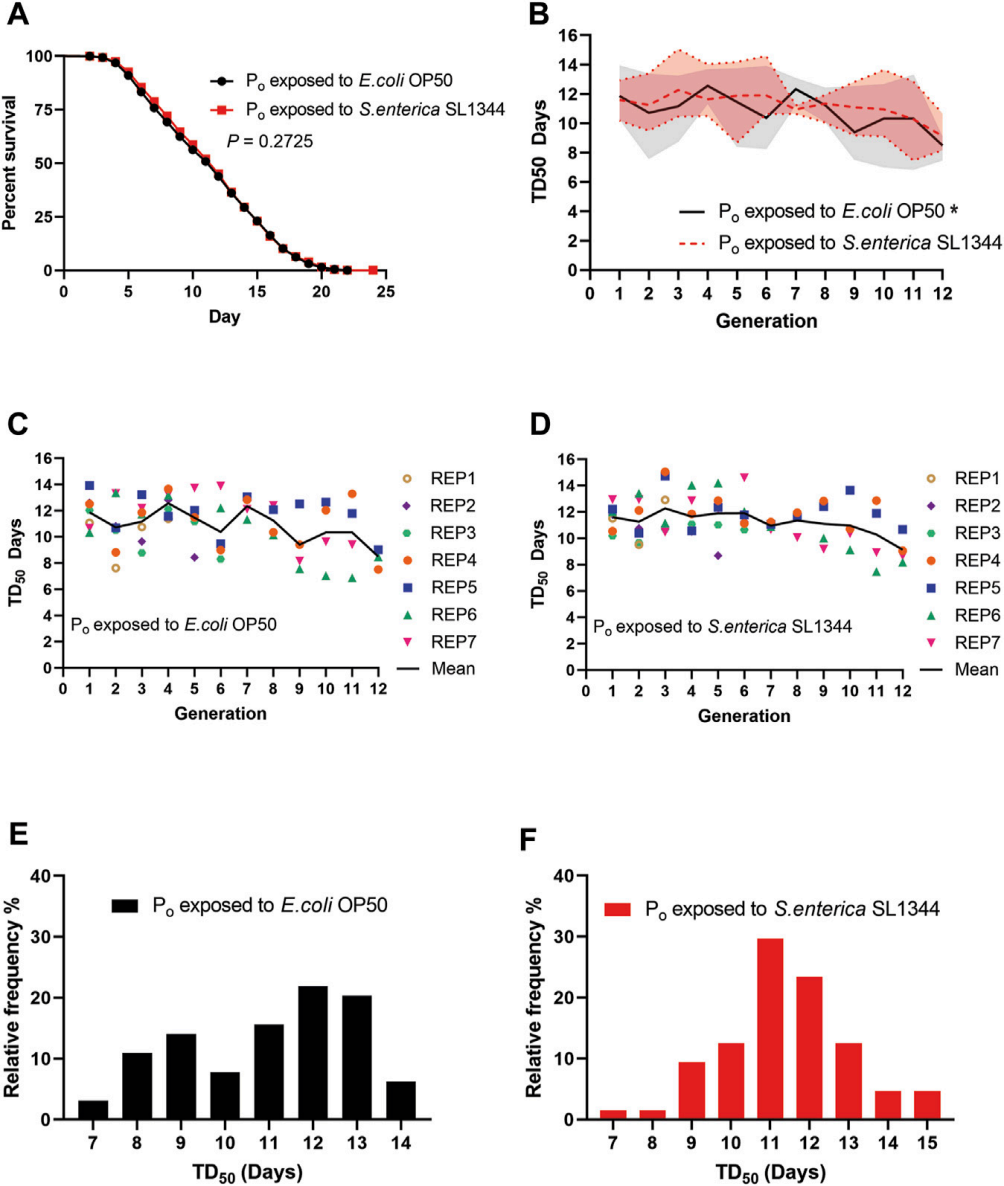
Pathogen infection during *C. elegans* development affects survival of subsequent generations. **(A)** Naïve and trained wild-type (WT) animals were exposed to *S. enterica* and scored for survival over time. The graph is a combination of 64 survival assays across seven independently generated naïve and trained lineages. *N* = 60 for each survival assay. *p-*value represents the significance level of survival between the two conditions, *p* = 0.2725. **(B)** The TD_50_ of each generation exposed to *S. enterica* and scored for survival was plotted over the respected generation. The graph is a combination of 64 survival assays across seven independently generated naïve and trained lineages. The trendlines represent the mean TD_50_ between seven independent replicates. The shaded area represents the TD_50_ range of the seven independent replicates. (*) denote a significant difference (*p* < 0.05). The *p*-values represent the significance level of a one-way ANOVA testing for differences between generations; naïve, *p* = 0.0313; trained, *p* = 0.2213. **(C,D)** The TD_50_ of each generation exposed to *S. enterica* and scored for survival was plotted over the respected generation. Each point represents the TD_50_ of a single survival assay, *N =* 60 for each survival assay. **(E,F)** The TD_50_ of the naïve and trained lineages were organized by their relative frequency and binned by 1-day increments. The histograms are a combination of 64 survival assays. *N* = 60 for each survival assay. The *p-*value represents the significance level of a Kolmogorov-Smirnov test to determine if the distributions of the naïve and trained lineage survival were statistically different, *p* = 0.3009.

Taking the average of all the survival assays, the trained animals did not show a significant difference in survival when exposed to *S. enterica* as compared to the naïve animals (Figure 2A). We then plotted the time that it took for 50% of the assay animals to die (TD_50_) for each generation against *S. enterica* to see if there were any differences between the two lineages on a generation-to-generation basis. When the TD_50_ of the naïve lineage was plotted over generations, the animals’ survival displayed a tendency to oscillate between generations forming a repeating pattern of peaks and valleys. This repeating pattern throughout the 12 generations was unexpected and has yet to be reported. A one-way ANOVA examining the TD_50_ values of the naïve lineage found a significant difference between generations. When the TD_50_ of the trained lineage was plotted and examined in the same manner, the oscillation was present but noticeably suppressed as compared to the naïve lineage and an ANOVA test found no significant difference between generations of the trained lineage (Figure 2B). The TD_50_ of all the survival assays for the naïve and trained lineages were also plotted alongside the means to better illustrate the data range (Figures 2C, D). Next, we examined the frequency distribution of all the survival assays. Though there was no significant difference between the two distributions, we do note that the naïve lineage had less defined peaks in frequency as compared to the trained lineage which centered around a single larger peak (Figures 2E, F). Taken together, these results demonstrate that TGIP does occur after an 8-h exposure to *S. enterica,* but the TGIP does not increase nor decrease survival on average as compared to the naïve lineage. Instead, the TGIP from the P_o_ generation stabilizes the later generations survival, reducing the generation-to-generation variability observed in the naïve lineage.

### 3.2 *S. enterica* exposure during development transgenerationally suppresses gene expression in *C. elegans*

Given the survival of the naïve lineage tended to fluctuate between generations as opposed to the trained lineage, we questioned if the expression of genes related to *S. enterica* infection were also fluctuating in the absences of pathogen infection. To this end, we collected uninfected samples of the two lineages at 56-h old, the same age as the P_o_ generation collection, and examined the relative gene expression of antimicrobial genes which are induced by *S. enterica* infection, *lipl-1,* and *lipl-3* (Xiao et al., 2017). Lipase-like 1 and 3 (*lipl-1, -3)* are predicted lipases located in lysosomes and expressed in the intestine of *C. elegans* (O'Rourke and Ruvkun, 2013). The *lipl* family of genes are regulated by MXL-3 and HLH-30, and control the utilization of internal energy reserves through autophagy and lipophagy (O'Rourke and Ruvkun, 2013). Since both *lipl-1* and *lipl-3* are upregulated during both *S. enterica* infection and food deprivation (Xiao et al., 2017; O'Rourke and Ruvkun, 2013), the stress response gene *gst-4* was also measured as a control. *gst-4* codes for the antioxidant enzyme Glutathione S-Transferase 4 and is induced by both starvation and pathogen infection (Hoeven et al., 2011; Tao et al., 2017), making it a well-suited indicator of false positives due to starvation or pathogen contamination.

**FIGURE 3:**
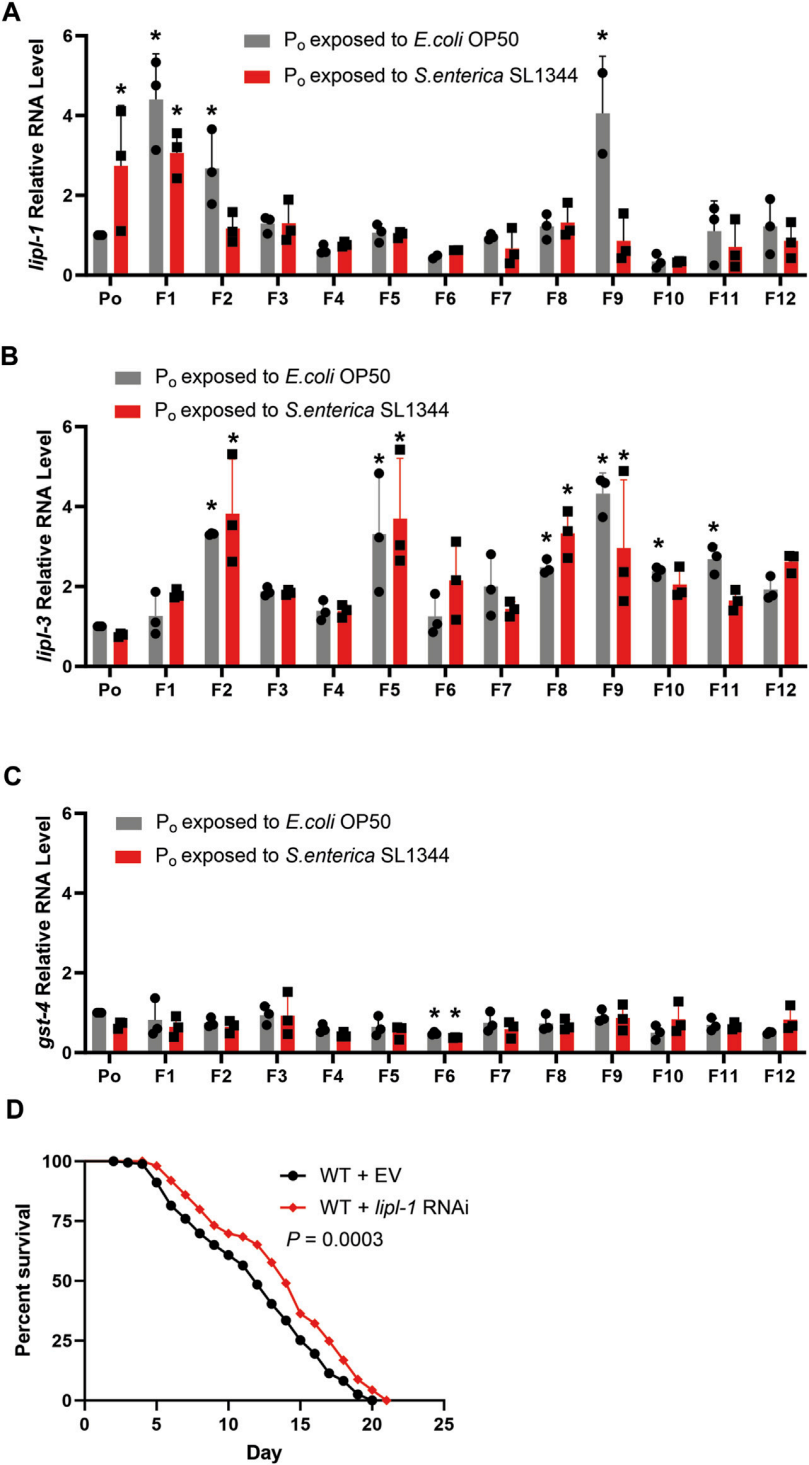
Antimicrobial response is attenuated by pathogen exposure in parental generations of *C. elegans* **(A)** qRT-PCR analysis of the naïve and trained WT animals prior to *S. enterica* exposure measuring *lipl-1*. **(B)** qRT-PCR analysis of the naïve and trained WT animals prior to *S. enterica* exposure measuring *lipl-3*. **(C)** qRT-PCR analysis of the naïve and trained WT animals prior to *S. enterica* exposure measuring *gst-4*. The graphs are a combination of RNA collections from three different replicates. Individual data points represent the relative quantity of each independently collected RNA sample normalized to pan-actin and the control sample. The bars graphs represent the mean, and the error bars represent the ±SD. Astericks (*) denote a significant difference (*p* < 0.05) in gene expression relative to P_o_ generation exposed to *E. coli*. Significance was determined by a one-way ANOVA and comparing the gene expression of each generation to the normalized expression of P_o_ generation exposed to *E. coli*, the reported *p*-value was adjusted using the Dunnett test to account for multiple comparisons. **(D)** Naïve animals grown on dsRNA for *lipl-1* or empty vector (EV) control were exposed to *S. enterica* and animal survival was scored over time. The graph is a combination of three independent replicates. *N* = 60 for each condition per replicate. *p-*value represents the significance level of survival between the EV control and the *lipl-1* treatment, *p* = 0.0003.

Quantifying the expression of *lipl-1, -3* and *gst-4* in the F_1_—F_12_ generations and comparing the RNA levels to the level in the P_o_ generation showed that the expression of *lipl-1* varied from generation to generation in the naïve lineage. By contrast, there is almost no variation in the expression of *lipl-1* in the trained lineage between generations F_2_ to F_8_ (Figure 3A). The expression of *lipl-3* however did fluctuate, regardless of lineage (Figure 3B). The F_9_ generation in the naïve population has an interesting spike in expression of *lipl-1* and *lipl-3,* reaching a mean relative quantity of 4.05 and 4.33 respectively. The cause of this spike during the F_9_ and the sudden drop in expression during the F_10_ is not clear. In neither lineage across all generations tested did the expression of *gst-4* significantly increase as compared to the P_o_ generation (Figure 3C), indicating that the increase of *lipl-1* expression in the naïve lineage was not due to a lack of food availability or pathogen contamination, but rather a possible innate fluctuation of gene expression. To confirm if *lipl-1* plays a role in survival against *S. enterica*, the naïve population was treated with *lipl-1* RNAi prior to pathogen challenge. Animals treated with *lipl-1* RNAi displayed an enhanced survival against *S. enterica* as compared to the empty vector control (Figure 3D). This enhanced survival phenotype of the *lipl-1* knockdown animals supports our previous observation that an overexpression of *lipl-1* is detrimental to the survival of *C. elegans* during *S. enterica* infection.

**FIGURE 4:**
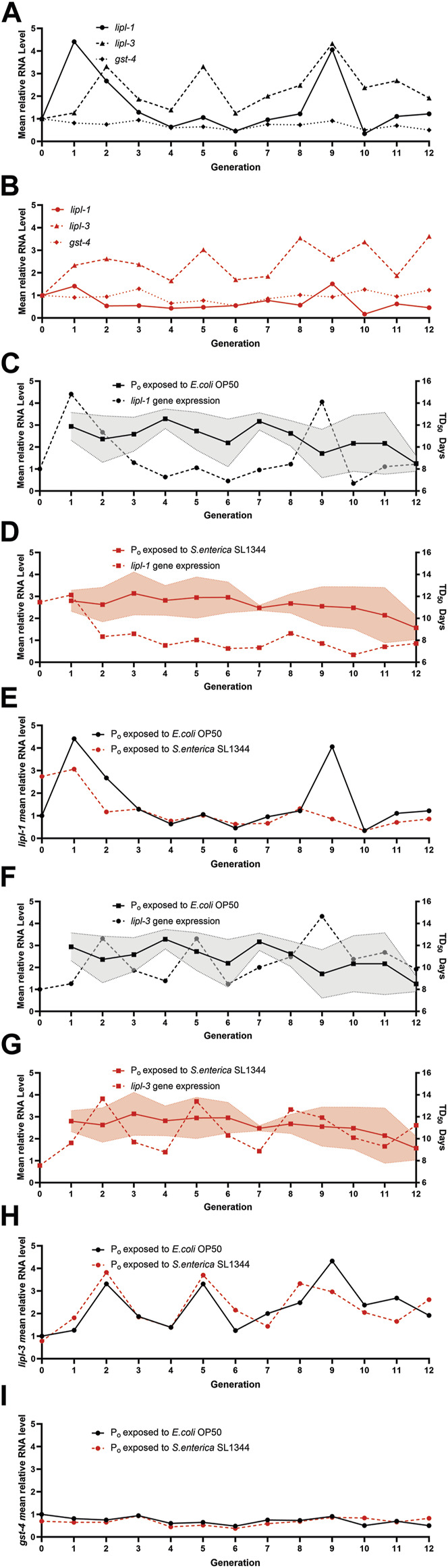
Transgenerational immune priming selectively suppresses gene expression. **(A)** *E. coli* exposed **(B)** *S. enterica* exposed lineages gene expression trends for *lipl-1, lipl-3,* and *gst-4.* Gene expression is relative to the respected P_o_ generation exposed to *E. coli* or *S. enterica*. **(C)** The *lipl-1* expression trend of the naïve lineages relative to P_o_ generation exposed to *E. coli* over 12 generations is plotted on the left *y*-axis while the survival tendency of the naïve lineages over 12 generations is plotted on the right *y*-axis. **(D)** The *lipl-1* expression trend of the trained lineages relative to P_o_ generation exposed to *E. coli* over 12 generations is plotted on the left *y*-axis while the survival tendency of the naïve lineages over 12 generations is plotted on the right *y*-axis. **(E)** The *lipl-1* expression trend over 12 generations of both the *E. coli* and *S. enterica* exposed lineages. Gene expression is relative to the respected P_o_ generation exposed to *E. coli*. **(F)** The *lipl-3* expression trend of the naïve lineages relative to P_o_ generation exposed to *E. coli* over 12 generations is plotted on the left *y*-axis while the survival tendency of the naïve lineages over 12 generations is plotted on the right *y*-axis. **(G)** The *lipl-3* expression trend of the trained lineages relative to P_o_ generation exposed to *E. coli* over 12 generations is plotted on the left *y*-axis while the survival tendency of the naïve lineages over 12 generations is plotted on the right *y*-axis. **(H)** The *lipl-3* expression trend over 12 generations of both the *E. coli* and *S. enterica* exposed lineages. Gene expression is relative to the respected P_o_ generation exposed to *E. coli*. **(I)** The *gst-4* expression trend over 12 generations of both the *E. coli* and *S. enterica* exposed lineages. Gene expression is relative to the respected P_o_ generation exposed to *E. coli*. The graphs are a combination of 3 independent replicates.

To better examine the expression of *lipl-1* and *lipl-3,* we plotted the mean relative quantity of each gene by generation to visualize any trends in expression (Figures 4A, B). Interestingly, when the mean relative quantity of *lipl-1* was plotted, the naïve generations with higher *lipl-1* expression tended to have a lower TD_50_ as compared to other generations (Figure 4C). In contrast, the expression of *lipl-1* in the trained lineages remained consistent between the F_2_—F_7_ generations, which correlates to the flattened survival tendency (Figures 4D, E). This near constant expression of *lipl-1* in the trained lineage falters starting on generation F_8_. Surprisingly, the two lineages share a similar expression trend with *lipl-3* which does not correlate to change in survival in either lineage (Figures 4F–H). Since *lipl-1* and *lipl-3* expression are regulated by the translocation of the transcription factor HLH-30 into the nucleus, it is assumed that translocation of HLH-30 would increase expression of both gene proportionally (O'Rourke and Ruvkun, 2013). It is unlikely that modifications to HLH-30 signaling are the cause of the *lipl-1* variation suppression. Comparing the expression of *gst-4* between the two lineages again showed no difference in expression over the course of 12 generations (Figure 4I). These results suggest a possible plasticity in the expression of genes involved in the innate immune response of *C. elegans.* Lineages without TGIP do not have the information to attenuate the expression of *lipl-1* and adjust its expression to their benefit. By contrast, the lineages with TGIP suppress *lipl-1* variation which correlates to a more consistent survival across multiple generations. However, TGIP does not suppress the whole HLH-30 pathway as *lipl-3* expression is unaffected after *S. enterica* exposure. These results point to a selective suppression of variation *lipl-1* that does not affect related genes such as *lipl-3*.

## 4 Discussion

In this study, we have shown that *C. elegans* exposed to *S. enterica* during development can pass information transgenerationally and prime the immune response of future offspring. Interestingly, the immune primer against *S. enterica* does not increase the average survival of the animals against the pathogen, but rather causes the individual survival times to become more consistent across generations. This consistency in survival across generations may be a response to increase the fitness of future generations in a diverse microbial environment similar to that of inherited avoidance behavior or increased expression of detoxification genes (Schulenburg and Félix, 2017; Ma et al., 2019; Moore et al., 2019; Burton et al., 2020). In the case of *S. enterica* infection, the primed animals suppressed the variation of *lipl-1* during development which may play a role in energy homeostasis before the addition of the pathogen stressor. Members of the lysosomal lipase family (*lipl-1, -2, -3, -5*) are regulated by the transcription factor HLH-30 and the suppressor MXL-3 during food abundant conditions (O'Rourke and Ruvkun, 2013). HLH-30, the nematode homolog of the mammalian protein Transcription Factor EB (TFEB), has been implicated in *C. elegans* survival against a variety of pathogen infections (Tsai et al., 2021; Wani et al., 2021; Goswamy et al., 2023).

Interestingly, we found that a spike in *lipl-1* expression correlated to a decrease in survival in the naïve lineage, most prominently during the F_9_ generation. This contrast previously reported data that a decrease in *lipl-1* expression decreases survival against *S. enterica* (Xiao et al., 2017). It is possible that both observations are valid as *lipl-1* may have a narrow homeostatic window due to it being a part of energy homeostasis and either suppression or overexpression may be detrimental to the animal’s survival. This phenomenon of a narrow homeostatic window has already been reported in *C. elegans* with the manipulation of *cep-1,* where knockdown and overexpression experiments of *cep-1* resulted in embryonic lethality (Derry et al., 2001).

While we showed the variation in *lipl-1* expression is lower in the trained lineage animals as compared to the naïve lineage, the same cannot be said for *lipl-3* for which both lineages have a similar expression pattern. Since *lipl-1* and *lipl-3* are shared targets for HLH-30, it is unlikely that change in expression is due to a change in HLH-30 translocation. Rather, it may be due to a modification of the histones packaging *lipl-1* or a change in DNA methylation which attenuates the expression of *lipl-1.* Further analyses of these epigenetic modifications are required to understand how animals regulate their gene expression and what pathways *S. enterica* infection stimulates during development to cause these changes.

Interestingly, during these experiments we observed a repeating pattern when measuring the survival of the naïve lineage. The TD_50_ of this lineage would periodically drop every 3–4 generations before recovering and surpassing the TD_50_ of the exposed lineage. Oscillation patterns especially in gene expression over development are well studied in *C. elegans*, but an oscillating pattern of survival in wild-type animals has yet to be reported and elaborated (Rashid et al., 2020). We hypothesize this repeating pattern is the result of an innate plasticity of *C. elegans* innate immune or stress response. During embryogenesis or gamete development *C. elegans* may tune its or its offspring’s epigenome in an attempt to increase their fitness. This rearrangement of gene regulation in a diverse environment to which *C. elegans* inhabits in the wild could serve as a means of countering unknown pathogens before exposure (Schulenburg and Félix, 2017). It is unclear to what extent this plasticity may help the organism survive, the mechanisms by which gene expression is modified, or if this is a pattern common among other *C. elegans* wild-type variants. These open questions will be pursued in our future studies.

In summary, we observed that exposure to *S. enterica* can invoke a change in transcription which affects survival in *C. elegans* and can last for at least 12 generations. This change can affect the expression of a gene but leave members of the same family of genes and transcription factor pathways unaffected. We also found that the survival of members of an unexposed lineage can have a repeating pattern of variation which points to a possible plasticity in immune and stress response between generations. Taken together, these results support studies that *C. elegans* can prime its offspring against specific pathogens to improve the fitness of later generations. Furthermore, the results show that TGIP does not always lead to an increase in the average survival time against a pathogen as compared to an untrain control. TGIP can instead stabilize the survival of a primed lineage, eliminating the valleys found in the unprimed lineage at the cost of the peaks. Flattening the survival tendency improves fitness by allowing primed offspring to outcompete unprimed animals exhibiting a drop in survival. The pattern of peaks and valleys in survival found in the unprimed lineage is also a point of interest. It may have gone unnoticed as studies using *C. elegans* often do not monitor survival over consecutive generations and pathogen infection in a parental generation can change this pattern. *C. elegans* may be utilizing a previously unaccounted epigenetic regulatory mechanism in the absence of priming pathogens. A system of possibly random adjustments to gene expression, not unlike random recombination of T cell receptors by the adaptive immune system (Krangel, 2009). This restructuring of gene expression at the epigenetic level could provide animals with only an innate immune system, a means to countering pathogens prior to exposure.

## Data Availability

The original contributions presented in the study are included in the article/Supplementary Material, further inquiries can be directed to the corresponding authors.
